# Commonly prescribed medications and risk of pneumonia and all-cause mortality in people with idiopathic pulmonary fibrosis: a UK population-based cohort study

**DOI:** 10.1186/s41479-024-00155-7

**Published:** 2025-01-25

**Authors:** Ann D. Morgan, Georgie M. Massen, Hannah R. Whittaker, Iain Stewart, Gisli Jenkins, Peter M. George, Jennifer K. Quint

**Affiliations:** 1 School of Public Health, Imperial College London, London, UK; 2 National Heart and Lung Institute, Imperial College London, London, UK; 3 Interstitial Lung Disease Unit, Royal Brompton Hospital and Harefield NHS Foundation Trust, London, UK

**Keywords:** Pneumonia, Idiopathic pulmonary fibrosis, Inhaled corticosteroids, Antacids, Proton pump inhibitors, Electronic health records

## Abstract

**Background:**

A growing body of evidence suggests that prolonged use of inhaled corticosteroids (ICS) and proton pump inhibitors (PPIs) is associated with increased risks of pneumonia. A substantial proportion of people with idiopathic pulmonary fibrosis (IPF) are prescribed PPIs or ICS to treat common comorbidities, giving rise to concerns that use of these medications may be associated with potential harms in this patient population.

**Methods:**

We used UK Clinical Practice Research Datalink (CPRD) Aurum primary care data linked to national mortality and hospital admissions data to create a cohort of people diagnosed with IPF on or after 1 January 2010. Patients were assigned to one of three exposure categories according to their prescribing history in the 12 months prior to IPF diagnosis as follows: “regular” users (≥ 4 prescriptions), “irregular” users (1–3 prescriptions) and “non-users” (no prescriptions). We explored the association between PPI/ICS prescription and pneumonia hospitalisation and all-cause mortality using multinomial Cox regression models.

**Results:**

A total of 17,105 people met our study inclusion criteria; 62.6% were male and 15.9% were current smokers. Median age at IPF diagnosis was 76.7 years (IQR: 69.6–82.7). 19.9% were regularly prescribed PPIs, and 16.0% ICS, prior to IPF diagnosis. Regular prescribing of PPIs and ICS was positively associated with hospitalisation for pneumonia; the adjusted HR for pneumonia hospitalisation comparing regular PPI users with non-users was 1.14 (95%CI: 1.04–1.24); for regular ICS users the corresponding HR was 1.40 (95%CI: 1.25–1.55). We also observed a small increased risk for all-cause mortality in the “regular ICS user” group compared with the “non-user” control group (HR_adj_ = 1.19, 1.06–1.33). We found no evidence of an association between PPI prescribing and all-cause mortality.

**Conclusion:**

Prolonged prescription of medications used to treat common comorbidities in IPF may be associated with increased risks for severe respiratory infections. These findings point to a need to adopt an adequate risk–benefit balance approach to the prescribing of ICS-containing inhalers and PPIs in people with IPF without evidence of comorbidities, especially older patients and/or those with more advanced disease in whom respiratory infections are more likely to result in poorer outcomes.

**Supplementary Information:**

The online version contains supplementary material available at 10.1186/s41479-024-00155-7.

## Introduction

Idiopathic pulmonary fibrosis (IPF) is a chronic interstitial lung disease with a poor prognosis [1]. It is characterised by progressive scarring of the lungs, and while rare in people under the age of 40 years, prevalence increases steeply with age [1]. IPF is more common in men than women, and although labelled as idiopathic, several factors including smoking and occupational exposures are thought to play a role in pathogenesis [2].

While the most common cause of death in people with IPF is respiratory failure due to IPF itself, around a third of patients die from comorbid conditions such as a cardiovascular disease or lung cancer [3]. Interest in the role played by comorbidities in IPF has increased in recent years, not just because comorbidities are very common in this typically older patient population [3, 4] but also because of concerns surrounding the potential to cause harm by either underdiagnosing and undertreating comorbidities or by inappropriate prescribing [5, 6]. In this context, medications used to treat chronic obstructive pulmonary disease (COPD) and gastro-oesophageal reflux disease (GORD) – namely inhaled corticosteroids (ICS) and proton pump inhibitors (PPIs) [7] – have garnered the most attention because of their associated pneumonia risks [8, 9]. The IPF-PPI debate is complicated by evidence that suggests that micro-aspiration caused by GORD is linked to IPF pathogenesis; this has led to the prescribing of antacids such as PPIs to IPF patients even in the absence of symptomatic GORD. However, studies exploring the associations between PPIs and mortality in IPF have shown inconsistent results, ranging from risk reductions of 50% to no association [10].

The evidence linking use of PPIs and ICS with increased risks for pneumonia is also mixed. For example, longer-term prescription of PPIs has been linked to an increased risk of incident pneumonia in a population-based cohort of individuals aged 60 years and over compared with age- and sex-matched controls not prescribed PPIs [11]. Previous studies have also demonstrated a positive association between PPIs and pneumonia in subgroups of people with diseases such as liver cirrhosis, type-II diabetes and stroke [12–14]. Among the COPD population, however, results are inconsistent, with some studies reporting increased risks [15] and others finding that PPI use did not contribute to a greater occurrence of pneumonia in people with COPD [16]. Whether PPI use increases pneumonia risks in the IPF population is a question that has not yet been addressed, but given the high prevalence of GORD among people with IPF (up to 70% according to some studies [3, 17]), it is one that merits an answer.

Much of the evidence linking use of ICS-containing inhalers with an increased risk of pneumonia stems from the COPD literature [18–21]. This evidence has recently been summarised by Miravitelles et al. in a comprehensive systematic review of both trial data and observational studies which concluded that exposure to ICS for ≥ 1 year increases the risk of pneumonia in COPD by 41% (RR = 1.41; 95%CI, 1.23–1.61) [22]. Such evidence, taken together with that from mechanistic studies which shows that the same mediators that are believed to drive the increased risk of pneumonia in people with COPD are also present in people with IPF [23–25], raises the prospect that prolonged use of ICS could also be harmful in IPF. This concern is heightened by the possibility that a proportion of people with IPF are being inappropriately treated with ICS, in the sense that they do not have comorbid COPD or asthma but have instead been misdiagnosed as having COPD and/or asthma in the period prior to their IPF diagnosis. Several studies have shown that misdiagnosis of IPF – which typically presents with non-specific symptoms such breathlessness and cough – is widespread [26].

In sum, few large-scale studies have specifically addressed the question whether use of PPIs or ICS increases pneumonia risk in people with IPF. The aims of this study were two-fold, firstly to examine the patterns of PPI and ICS prescribing among a UK population-based cohort of people with a diagnosis of IPF (with and without comorbid COPD and GORD) using routinely-collected primary and secondary healthcare data, and secondly to test the hypothesis that people with IPF prescribed PPIs or ICS on a regular basis are at increased risk for severe pneumonia or all-cause mortality.

## Methods

### Data source

Primary care data were provided by the Clinical Practice Research Datalink (CPRD). We used CPRD’s Aurum database (February 2022 build), which contains routinely-collected GP data for around 20% of the UK population. These data were linked to Hospital Episode Statistics (HES), specifically inpatient (admitted patient care or APC) data, mortality data curated by the Office for National Statistics (ONS), and socioeconomic data (Index of Multiple Deprivation (IMD)). These linkages were provided by CPRD for the purposes of this study but were only possible for English practices. CPRD Aurum data are representative of the population of England, with respect to age, sex, location and socioeconomic status [27].

### Study population

To be included in the study, people had to have received their diagnosis of IPF between 1 January 2010 and 31 December 2019 and be aged 40 years or over at the time of IPF diagnosis. The code set that was used to define our cohort of people with IPF was developed and validated by the authors of this study [28]. Diagnosis date was the date of the first IPF record in primary care. Study participants also had to meet certain data quality standards, be male or female, and be registered at a GP practice eligible for linkage to HES and ONS data. Finally, participants had to have at least one year of continuous registration with the same GP prior to their IPF diagnosis; this one-year period of registration was used to categorise participants into one of three exposure categories based their medication history (see below) and was chosen to achieve a balance between adequate sample size and a sufficiently long period for defining longer-term use. People were followed up until the earliest of: the end of the study period (31/12/2019), date of death, date of transfer out of practice, last collection date of practice data, or date of last HES and ONS data collection (see Supplementary Fig. S1).

### Exposure definition

We defined two exposures of interest, prescription of PPIs or ICS. People were categorised into three exposure groups, depending on the number of prescriptions in the 12 months prior to their IPF diagnosis (baseline period). Those who were prescribed either a PPI or an ICS-containing inhaler on four or more occasions (at least 1 month apart) were classified as “regular” users; those who had 1–3 prescriptions were described as “irregular” users, and those with no evidence of a prescription in the baseline period were categorised as “non-users” (control group). These categorisations were based on clinical experience and knowledge of UK GP prescribing practices and are consistent with the approach adopted by other studies. For the purposes of this study, we defined ICS inhalers as any ICS-containing inhaler approved by NICE for the treatment of COPD or asthma, i.e. ICS monotherapy inhalers, combination inhalers (ICS with long-acting beta-agonists (ICS + LABA) or ICS with long-acting muscarinic antagonists (ICS + LAMA)) and triple therapy inhalers (ICS + LABA + LAMA).

### Outcomes

Our main outcome of interest was hospitalisation for pneumonia during follow up (first and subsequent events). Hospitalisations for pneumonia were identified in APC HES data using ICD-10 codes (J12.xx–J18.xx). We only included events in which the ICD-10 pneumonia code was listed as the primary diagnosis in order to exclude occurrences of hospital-acquired pneumonia. Our secondary outcome was all-cause mortality, as recorded in ONS data.

### Covariates

Demographic patient characteristics (sex, age at IPF diagnosis) were obtained from primary care data; socioeconomic status (quintiles) was extracted from linked IMD records. People were categorised into four age bands, 40–59, 60–69, 70–79, 80 + years. Lifestyle characteristics [body mass index (BMI (underweight, normal, overweight, obese), smoking status (current, ex, never)] were determined at the time of IPF diagnosis (using the closest recorded values to this date). The presence of the following comorbidities at any point prior to IPF diagnosis was described using a binary variable (yes, no): COPD, asthma, pulmonary arterial hypertension, lung cancer, GORD, ischaemic heart disease (IHD), hiatus hernia, heart failure and diabetes. Prescription of oral corticosteroids in the baseline period was also described using a binary variable (PPI analysis).

All codelists used in this study can be accessed from: https://github.com/NHLI-Respiratory-Epi/IPF-PPI-ICS.

### Data analyses

#### Descriptive analysis

We explored patterns of ICS and PPI use in our study cohort, comparing the frequency of prescribing in the 12-months prior to IPF diagnosis with that in the 12-month period after. Baseline covariates (as specified above) were reported for the cohort as a whole and separately for our two exposed groups (regular and irregular users) and our control group (non-users), using frequencies for categorical variables and medians for continuous variables.

#### Statistical analysis

Cox proportional hazard models were implemented to estimate hazard ratios (HRs) for the time to first pneumonia hospitalisation and all-cause mortality respectively, comparing non-users with irregular and regular users, separately for PPI and ICS. We used Schoenfeld residuals to check that proportional hazard assumptions were met. Rates of pneumonia hospitalisation were estimated using a negative binomial model.

We utilised an intention-to-treat methodology such that people remained in the exposure groups they were assigned at baseline. However, as part of a series of sensitivity analyses, we repeated our primary analysis (hospitalisation for pneumonia) but excluded individuals who were prescribed ICS only after IPF diagnosis from our “non-users” control group. We also investigated, among those who were hospitalised due to pneumonia, how many were still prescribed PPIs in the 3 months prior to their hospitalisation. In the context of assessing confounding by indication, in addition to adjusting for co-diagnoses of COPD and/or asthma in the case of the ICS analysis, and GORD and hiatus hernia in the case of the PPI analysis, we also tested for effect modification, and where this was significant reported stratified HRs for the association between medication use and our outcomes (pneumonia hospitalisation and all-cause mortality) for people with and without the corresponding comorbidities.

All analyses were performed using Stata version 17. StataCorp LLC, TX, USA.

## Results

### Cohort characteristics and prescribing patterns

A total of 17,105 people met our study inclusion criteria (Supplementary Fig. S2); 62.6% were male and 15.8% were current smokers. Median age at IPF diagnosis was 76.7 years (IQR: 69.6–82.7). Nearly a quarter (23.1%) had a GP-recorded diagnosis of GORD prior to their IPF diagnosis, while 16% had a prior diagnosis of COPD. A similar proportion (17%) had been previously diagnosed with asthma. IHD, pulmonary hypertension, diabetes also ranked as highly prevalent comorbidities in our IPF study population (relative to the general adult population) (Table 1).

**Table 1:** Baseline characteristics of study cohort

Characteristic	Whole cohort	PPI			ICS		
		**Regularly prescribed**	**Irregularly prescribed**	**Not prescribed**	**Regularly prescribed**	**Irregularly prescribed**	**Not prescribed**
	**n (% of total)**^**a**^	**n (% of total)**^**a**^	**n (% of total)**^**a**^	**n (% of total)**^**a**^	**n (% of total)**^**a**^	**n (% of total)**^**a**^	**n (% of total)**^**a**^
**Total**	17,105 (100.0%)	3,396 (19.9%)	1,649 (9.6%)	12,060 (50.5%)	2,741 16.0%)	1,507 (8.8%)	12,857 (75.2%)
***Sex***							
Male	10,706 (62.6)	2,058 (60.6%)	1,000 (60.6%)	7,648 (63.4%)	1,584 (57.8%)	894 (59.3%)	8,228 (64.0%)
Female	6,399 (37.4)	1,338 (39.4%)	649 (39.4%)	4,412 (36.58%)	1,157 (42.2%)	613 (40.9%)	4,629 (36.0%)
***Age (years)***							
Median (IQR)	76 (69–83)	77 (70– 83)	76 (69—83)	77 (70– 83)	76 (68 –81)	75 (67–81)	77 (70–83)
40–59	1,263 (7.4%)	188 (5.5%)	129 (7.8%)	859 (7.12%)	241 (8.8%)	148 (9.8%)	874 (6.8%)
60–69	3,214 (18.8%)	598 (17.6%)	322 (19.5%)	2,147 (17.8%)	570 (20.8%)	358 (23.8%)	2,286 (17.8%)
70–79	6,516 (38.1%)	1,304 (38.4%)	585 (35.5%)	4,489 (37.22%)	1,104 (40.3)	546 (36.2%)	4,866 (37.9%)
> = 80	6,112 (35.7%)	1,306 (38.5%)	613 (37.2%)	4,565 (37.9%)	826 (30.1%)	455 (30.2%)	4,831 (37.6%)
***Index of Multiple Deprivation***							
1 (least deprived)	3,530 (20.7%)	658 (19.4%)	301 (18.3%)	2,571 (21.3%)	458 (16.7%)	319 (21.2%)	2,753 (21.4%)
2	3,676 (21.5%)	675 (19.9%)	345 (21.0%)	2,656 (22.0%)	509 (18.6%)	307 (20.4%)	2,860 (22.2%)
3	3,204 (18.8%)	611 (18.0%)	312 (19.0%)	2,281 (18.9%)	492 (18.0%)	304 (20.2%)	2,408 (18.7%)
4	3,211 (18.8%)	659 (19.5%)	313 (19.0%)	2,239 (18.6%)	561 (20.5%)	265 (17.6%)	2,385 (18.6%)
5 (most deprived)	3,464 (20.3%)	785 (23.2%)	375 (22.7%)	2,304 (19.1%)	713 (26.1%)	311 (20.6%)	2,440 (19.0%)
Missing	20 (0.1%)	8 (0.2%)	3 (0.2%)	9 (0.1%)	8 (0.29%)	1 (0.07%)	11 (0.09%)
***Body mass index (kg/m***^***2***^***)***							
Underweight	466 (2.7)	72 (2.1%)	41 (2.5%)	353 (2.9%)	79 (2.9%)	47 (3.1%)	340 (2.6%)
Normal (baseline)	3,775 (22.1)	710 (20.9%)	395 (24.0%)	2,671 (22.1%)	641 (23.4%)	313 (20.8%)	2,821 (21.9%)
Overweight	4,502 (26.3)	969 (28.5%)	438 (26.6%)	3,095 (25.7%)	771 (28.1%)	400 (26.5%)	3,331 (25.9%)
Obese	3,399 (19.9)	723 (21.3%)	294 (17.8%)	2,122 (17.6%)	705 (25.7%)	373 (24.8%)	2,321 (18.1%)
Missing	4,963 (29.0)	922 (27.2%)	481 (29.2%)	3,820 (31.7%)	545 (19.9%)	374 (24.8%)	4,044 (31.5%)
***Smoking status***							
Never	1,950 (11.4%)	335 (9.9%)	185 (11.2%)	1,430 (11.9%)	218 (8.0%)	173 (11.5%)	1,559 (12.1%)
Ex-smoker	12,431 (72.7%)	2,558 (75.3%)	1,221 (74.0%)	8,667 (71.8%)	2,024 (73.8%)	1,091 (72.4%)	9,316 (72.5%)
Current smoker	2,712 (15.8%)	503 (14.8%)	243 (14.8%)	1,951 (16.2%)	499 (18.2%)	243 (16.1%)	1,970 (15.3%)
Missing	12 (0.1%)	0 (0.0%)	0 (0.0%)	12 (0.1%)	0 (0.0%)	0 (0.0%)	12 (0.1%)
***Comorbidities***							
GORD	3,957 (23.1%)	1,330 (39.2%)	527 (68.0%)	2,100 (17.4%)	763 (27.8%)	372 (24.7%)	2,822 (22.0%)
COPD	2,777 (16.2%)	654 (19.26%)	276 (16.7%)	1,847 (15.3%)	1,396 (50.9%)	384 (25.5%)	997 (7.8%)
Asthma	2,911 (17.0%)	695 (20.5%)	291 (17.7%)	1,925 (16.0%)	1,662 (60.6%)	481 (31.9%)	768 (6.0%)
PAH	313 (1.8%)	66 (1.6%)	29 (1.8%)	218 (1.8%)	56 (2.0%)	25 (1.7%)	232 (1.8%)
Lung cancer	137 (0.8%)	28 (0.85%)	15 (0.9%)	94 (0.8%)	32 (1.2%)	11 (0.7%)	94 (0.7%)
IHD	4,657 (27.2%)	1,242 (36.6%)	493 (29.9%)	2,922 (24.2%)	735 (26.8%)	387 (25.7%)	3,535 (27.5%)
Hiatus hernia	2,445 (14.3%)	894 (26.3%)	293 (17.8%)	1,258 (10.4%)	456 (16.6%)	212 (14.1%)	1,777 (13.8%)
Heart failure	1,844 (10.8%)	423 (12.5%)	182 (11.0%)	1,239 (10.3%)	284 (10.4%)	117 (7.8%)	1,443 (11.2%)
Diabetes	4,052 (23.7%)	908 (26.7%)	416 (25.2%)	2,728 (22.6%)	633 (23.1%)	357 (23.7%)	3,062 (23.8%)

*COPD* Chronic obstructive pulmonary disease, *GORD* Gastro-oesophageal reflux disease, *ICS* Inhaled corticosteroid, *IHD* Ischaemic heart disease, *PAH* Pulmonary arterial hypertension, *PPI* Proton pump inhibitor

^a^Unless otherwise specified

Of the 17,105 people with IPF, 5,045 (29.5%) were prescribed a PPI at least once in the year prior to their IPF diagnosis, of whom 1,857 (36.8%) also had a diagnosis of GORD (Table 1). Of the people also diagnosed with hiatus hernia (*n = *2,445), 48.5% were prescribed a PPI in the baseline period. Among those not prescribed a PPI prior to their IPF diagnosis, 1,380 went on to receive a prescription for PPI in the year after their diagnosis, but were classed as “non-users”, and contributed data to the control population (Table 2).

**Table 2:** PPI prescribing patterns pre- and post-IPF diagnosis

		**Prescription in year post IPF diagnosis**			
		**No PPI**	**Irregular PPI**	**Regular PPI**	**Total**
**Prior prescription**	**No PPI**	10,680	900	480	12,060
	**Irregular PPI**	639	528	482	1,649
	**Regular PPI**	228	609	2,559	3,396
	**Total**	11,547	2,037	3,521	17,105

*IPF* Idiopathic pulmonary fibrosis, *PPI* Proton pump inhibitor

**Fig. 1:**
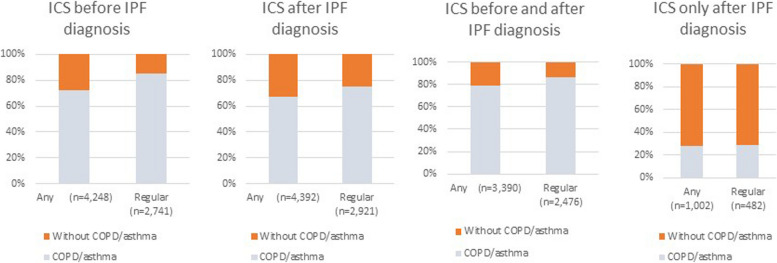
Patterns of ICS prescribing pre- and post-IPF diagnosis. ICS: Inhaled corticosteroids, COPD: chronic obstructive pulmonary disease

Regular and irregular ICS users comprised 16% (*n = *2,741) and 8.8% (*n = *1,507) of the study cohort, respectively (Table 1). Unsurprisingly, patients with a prior diagnosis of COPD or asthma were overrepresented in the ICS user groups, especially the regular users. Among the prior non-ICS users (*n = *12,857), 1,002 (or around 6% of the whole cohort) were prescribed an ICS-containing inhaler at least once after their IPF diagnosis (Fig. 1). Nearly a half of this subgroup (*n = *482; 48.1%) became regular ICS users post IPF diagnosis. Whereas patients who also had COPD and/or asthma dominated the pre-, post- and pre-and-post IPF ICS user groups (Fig. 1a, b and c, respectively), among those who started on ICS only after an IPF diagnosis, only a third also had either a previous COPD or asthma diagnosis (Fig. 1d). Among the subset of 1,002 new users, 719 (71.8%) were prescribed ICS at least once after their IPF diagnosis without having a concomitant diagnosis of either COPD or asthma, and 342 (34.1%) became regular ICS users (Supplementary Table S1). Since only a very low proportion of the cohort (*n = *188; 1.1%) were regularly prescribed both ICS and PPIs in the baseline period, we did not mutually adjust for ICS and PPI use in our subsequent statistical analyses.

### Pneumonia hospitalisation

A total of 3,651 people with a diagnosis of IPF were admitted to hospital on at least one occasion for treatment for pneumonia, of whom 770 (21.1%) were regularly prescribed PPI and 391 (10.7%) were irregularly prescribed PPI in the year prior to IPF diagnosis. Similarly, 770 (21.1%) of the people hospitalised with pneumonia had a history of regular ICS prescription; 30.1% of those hospitalised had been prescribed ICS at least once.

**Fig. 2:**
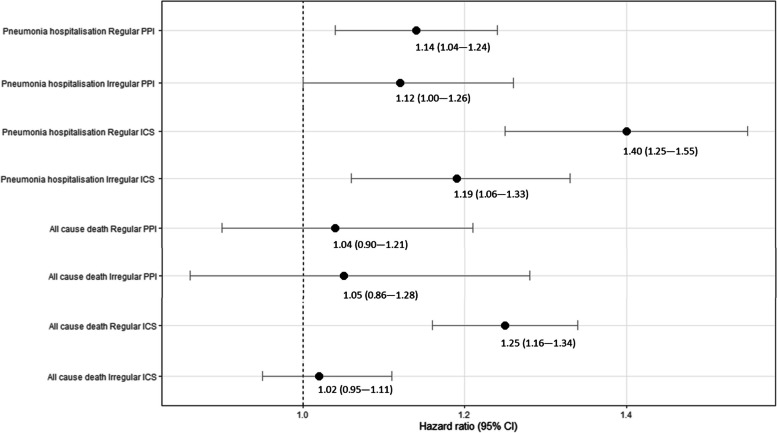
Hazard ratios for pneumonia hospitalisation and all-cause mortality relative to prescriptions of PPI and ICS in a cohort of people with IPF. PPI: proton pump inhibitor, ICS: inhaled corticosteroids

In adjusted Cox regression analyses, any prescription of PPIs and ICS in the 12-month period prior to IPF diagnosis was associated with a modest increased risk of hospitalisation for pneumonia. In the case of ICS, there was some evidence of a dose–response relationship (Fig. 2; Supplementary Table S2). The adjusted rate of hospitalisations for pneumonia was also elevated in patients who were prescribed PPIs in the baseline period (relative to non-users) (Table 3). The association was more pronounced in ICS users; hospitalisations were increased by 51% in regular ICS users (95% CI: 33%–72%) and by 26% in irregular users (95% CI: 10%–44%), compared with non-users.

**Table 3:** Association between medication use and rate of pneumonia hospitalisations in a cohort of people with IPF (complete-case analysis)

	**Unadjusted model**			**Fully-adjusted model**^**a**^		
	**IRR**	**95% CI**	***P*-value**	**IRR**	**95% CI**	***P*-value**
***Proton pump inhibitors***						
Non-users	0.00			0.00		
Irregular users	1.24	1.08–1.43	0.002	1.18	1.03–1.35	0.018
Regular users	1.22	1.10–1.35	< 0.001	1.14	1.03–1.26	0.012
***Inhaled corticosteroids***						
Non-users	0.00			0.00		
Irregular users	1.28	1.12–1.47	< 0.001	1.26	1.10–1.44	0.001
Regular users	1.79	1.62–1.98	< 0.001	1.51	1.33–1.72	< 0.001

*CI* Confidence interval, *IRR* Incident rate ratio

^a^Adjusted for age, sex, smoking history, index of multiple deprivation, COPD, asthma, lung cancer, gastro-oesophageal reflux disease, hiatus hernia, ischaemic heart disease, heart failure, pulmonary artery hypertension and type 2 diabetes. In the case of the PPI analysis, the fully-adjusted model was additionally adjusted for prescription of an oral corticosteroid (at least once in the 12 months prior to IPF diagnosis)

### All-cause mortality

Among our study cohort, all of whom were diagnosed with IPF after 1/1/2010, around a half (*n = *8,964; 52.4%) died before the end of the study period (31/12/2019). The median survival time for this group of patients was 1.7 years (IQR: 0.68–3.25). Prescription of PPIs (regularly or irregularly) in the baseline period was not significantly associated with all-cause mortality (Fig. 2). In a crude Cox regression analysis, we found that people who were prescribed ICS on a regular basis had a small increased risk of dying from any cause relative to non-ICS users (HR_unadj_ = 1.06; 95%CI, 1.03–1.25) (Table S2). Adjustment for both demographic/lifestyle factors and comorbidities had the effect of increasing the HR for this association (HR_adj_ = 1.25; 95%CI, 1.16–1.34), reflecting the tendency in this IPF cohort towards a higher prevalence of comorbidities such as COPD in the ICS-user group compared with the non-users (Table 1). Irregular use of ICS was not associated with a shorter survival time, relative to non-use, in either a crude analysis or adjusted analysis (Table S2).

### Sensitivity analyses

Details of our sensitivity analyses are provided in Supplementary Material 1. In summary, we found that in the case of our PPI analysis neither GORD nor hiatus hernia were effect modifiers of the association between PPI prescribing and our primary and secondary outcomes (hospitalisation for pneumonia and all-cause mortality). We found only weak evidence to suggest that concomitant diagnoses of COPD or asthma had a modifying effect on the relationship between ICS prescribing and risk of pneumonia hospitalisation (see Supplementary Tables S3–S4).

## Discussion

In a cohort of patients diagnosed with IPF between 2010 and 2019, we observed a positive association between prescription of ICS in the year prior to diagnosis and hospitalisation for pneumonia, which remained significant after adjustment for key confounders. We found a similar, slightly weaker relationship, for PPI. We also observed a small increased risk for all-cause mortality in the “regular ICS user” group (compared with the “non-user” control group); however, we found no evidence of an association between the prescription of PPIs and all-cause mortality.

While other studies have reported increased pneumonia risks among longer-term users of ICS and PPIs, this is one of the largest studies to explicitly address this question in an IPF population. Our findings suggest that the same risks are present in people with IPF, which – given their age and high prevalence of conditions which are indications for ICS and PPIs – should be viewed as a potential cause for concern. At the very least the evidence for increased pneumonia risk should prompt clinicians to carefully consider the risk–benefit balance of continuing (or initiating) ICS/PPI use in people newly-diagnosed with IPF. This may be especially pertinent in people whose presenting respiratory symptoms may have been misdiagnosed as COPD or late-onset asthma prior to their IPF diagnosis, and in whom continuation of ICS is unlikely to confer any benefit. Several studies have established that misdiagnosis of non-specific respiratory symptoms is not only common in IPF but also leads to diagnostic delays, of up 5 years in some cases, as well as poorer outcomes [29, 30].

In our study cohort, around three quarters of patients who were prescribed ICS prior to their IPF diagnosis continued to be prescribed ICS post-diagnosis. Both prior- and post-ICS prescription was highly correlated with a prior diagnosis of either COPD or asthma, but we were not able to ascertain what proportion of our study cohort had comorbid COPD or asthma and what proportion had been misdiagnosed and therefore potentially inappropriately treated. Nevertheless, given estimates of the proportion of IPF patients with comorbid COPD/emphysema tend to centre around 30% [3], we suspect that a sizeable number of IPF patients may be continuing to be prescribed ICS-containing inhalers post diagnosis even though they do not have confirmed obstructive disease.

In contrast, we found little evidence of over-prescribing of PPIs in our study cohort. The IPF management guidelines that were in place during the period of this study [31, 32] include a conditional recommendation to consider treating people with IPF with antacid therapies, irrespective of GORD symptoms. This was based on early retrospective data demonstrating improved survival [33] and a slowing of disease progression [34] in people with IPF on antacids. Subsequent studies, which found no longer-term benefit of antacids and in one case evidence of increased risk of respiratory infections in patients with advanced IPF [35], have brought this recommendation into question [36–38] and the latest (2022) guidelines instead advise clinicians to only prescribe antacid medication to treat symptoms of GORD when present [39]. We found that only 20.6% of our cohort were prescribed PPIs on at least four occasions in the year following their diagnosis, suggesting that even during the period that they were active, the older guidelines were not necessarily being followed.

While in our study cohort both ICS and PPI prescribing was associated with an increased risk for pneumonia, only those prescribed ICS on a regular basis appeared to be at increased risk for all-cause mortality. We observed a statistically significant 25% increase in risk for all-cause mortality in regular ICS users, after adjustment for age, smoking status and comorbid conditions including COPD and lung cancer. The reasons behind this apparent association are likely complex, but without further investigation, we can only speculate as to the possible drivers. Part of the increased mortality risk might be attributed to higher likelihood of diagnostic delays in those prescribed inhaled therapies prior to their IPF diagnosis, denying patients the opportunity to initiate anti-fibrotic therapies at an earlier disease stage. Support for this hypothesis comes from a study conducted by Hoyer et al. involving 264 patients which found that longer diagnostic delays (over 1 year) were associated with worse progression-free survival times than shorter delays (< 1 year) (HR = 1.70, 95% CI: 1.18–2.46, *p* = 0.004). Interestingly, diagnostic delay was not significantly associated with all-cause survival (HR = 1.54, 95% CI: 0.95–2.51, p = 0.08) but the small sample size may mean that this study was underpowered with respect to mortality [30].

### Strengths and limitations

The main strength of this study derives from the use of CPRD’s Aurum dataset to define a large population-based cohort of people with a diagnosis of IPF. Validation work has confirmed the high positive predictive value (PPV) of GP-recorded diagnoses of IPF in this database [28]; this primary-care dataset has the added advantage of containing accurate prescribing information. Nevertheless, we have had to rely on prescribing history as a proxy for medication use as Aurum does not provide data on whether prescriptions were filled; this may result in overestimation of actual medication use. Conversely, over-the-counter antacid therapies have not been captured, and it is therefore possible that we have underestimated PPI use. However, for a high proportion of our study cohort, prescriptions are free of charge (i.e. in the over 60s) and thus use of over-the-counter antacids may be less significant.

Our study has a number of other limitations. Identification of our primary outcome events, hospitalisation for pneumonia, relies on diagnosis and ICD-10 coding by hospital consultants and staff, and we cannot be certain that diagnoses have been confirmed by microbiological testing. It is also likely that there is some misclassification of pneumonia outcomes, with some events in truth being acute exacerbations of IPF. Between 10% and 20% of people with IPF experience an acute exacerbation each year and such events are associated with high mortality [40]. However, misclassification is most likely non-differential and as such would have minimal impact on our Cox-regression-based conclusions.

This being an observational study, establishing cause-and-effect can be challenging and we acknowledge that we may not have fully adjusted for all potential sources of confounding. In the context of this study, confounding by indication is likely to be a significant issue. Adjustment for COPD and asthma did indeed noticeably reduce the magnitude of our HRs, but even after adjustment for these conditions, ICS use – even irregular use – was strongly associated with an increased risk of severe pneumonia, from which we inferred that while clearly present, confounding by indication alone cannot account for our findings. Furthermore, our sensitivity analyses, in which we explored the possibility of effect modification by COPD and/or asthma, found little evidence to suggest that the presence of COPD/asthma was driving the observed association: our stratified analyses indicate increased risks for both outcomes irrespective of the presence or absence of COPD and/or asthma (see Supplementary Material 1). The same is true of the PPI analyses: we found no evidence of effect modification of the association between PPI use and pneumonia by the presence of GORD or hiatus hernia. That said there may well be other risk factors for pneumonia which are also associated with the ICS and PPI use that we have not been able to account for (e.g. previous infections, more severe disease).

While we acknowledge that use of a time-varying exposure may have been a more robust modelling strategy, we considered that in our cohort this approach was not warranted and unlikely to materially affect our conclusions. As part of our descriptive analyses, we ascertained that most people who were prescribed ICS before their IPF diagnosis continued to be prescribed ICS after. A sensitivity analysis in which we excluded those individuals who only initiated ICS therapy after IPF diagnosis from the control group generated broadly similar results. In addition, we determined that most people who were prescribed PPI in the baseline exposure period were also prescribed PPI in the 3 months prior to their first pneumonia hospitalisation.

Finally, we were unable to explore what may be driving the observed increased risk for all cause-mortality in regular ICS users. However, this question merits further study, especially as this finding appears to be peculiar to the IPF patient population. In people with COPD, ICS use is not generally associated with increased mortality and is likely to be protective in some subgroups, such as frequent exacerbators and those with concomitant asthma. Replication of our study in other IPF cohorts would confirm whether an increased mortality risk is unique to our study cohort, which with a survival time of 1.7 years from IPF diagnosis, is likely to be dominated by individuals with more advanced IPF.

## Conclusion

We have shown that prolonged prescription of medications used to treat COPD and GORD, both common comorbidities in IPF, may be associated with increased risks for severe respiratory infections. While as yet unconfirmed in other IPF cohorts, these findings point to a need to adopt an adequate risk–benefit balance approach to the prescribing of ICS-containing inhalers and PPIs in people with IPF without evidence of these comorbidities, especially older patients and/or those with more advanced disease in whom respiratory infections are more likely to result in poorer outcomes, hospitalisation and even death. While our PPI findings add to the ongoing debate over the safety and efficacy of antacid therapy in IPF [41], they do not provide resolution and further studies, including appropriately designed clinical trials, are needed. We await the results of the ongoing TIPAL trial of lansoprazole in IPF with interest (ISRCT Number: ISRCTN13526307).

## Supplementary Information

Supplementary Material 1.

## Data Availability

Code sets used in this study are available to download from the GITHub repository (https://github.com/NHLI-Respiratory-Epi/IPF-PPI-ICS).

## Abbreviations

COPD: Chronic obstructive pulmonary disease
CPRD: Clinical Practice Research Datalink
GORD: Gastro-oesophageal reflux disease
HES: Hospital Episode Statistics
ICD-10: International Classification of Disease (10th revision)
ICS: Inhaled corticosteroid
IPF: Idiopathic pulmonary fibrosis
LABA: Long-acting beta-agonist
LAMA: Long-acting muscarinic antagonist
NICE: National Institute for Clinical Excellence
ONS: Office of National Statistics
PPI: Proton pump inhibitor
