# Supplementary material for: Commonly prescribed medications and risk of pneumonia and all-cause mortality in people with idiopathic pulmonary fibrosis: a UK population-based cohort study
Source: Pneumonia (Nathan). 2025 Jan 25;17:2. doi: 10.1186/s41479-024-00155-7 (PMC11762896; doi:10.1186/s41479-024-00155-7)
Supplement: Supplementary file 1 — Supplementary Material 1. [file 41479_2024_155_MOESM1_ESM.docx]

**SUPPLEMENTARY MATERIAL**

**Title**: Commonly prescribed medications and risk of pneumonia and all-cause mortality in people with idiopathic pulmonary fibrosis: a UK population-based cohort study

**Authors**: Ann D Morgan^1^, Georgie May Massen^1^, Hannah R Whittaker^1^, Iain Stewart^2^, Gisli Jenkins^2^, Dr Peter George^3^, Jennifer K Quint^1^

**Affiliations**:

1. School of Public Health, Imperial College London, London, UK

2. National Heart and Lung Institute, Imperial College London, London, England

3. Interstitial Lung Disease Unit, Royal Brompton Hospital and Harefield NHS Foundation Trust, London, UK

Corresponding author: [a.morgan15@imperial.ac.uk](mailto:a.morgan15@imperial.ac.uk)

List of items

**Fig. S1** | Study design (definition of the index date)

**Fig. S2** | Study cohort (patient flow chart)

**Table S1** | Patterns of ICS prescribing, pre- and post-IPF diagnosis

**Table S2 |** Association between PPI/ICS use and pneumonia hospitalisation and all-cause mortality in a cohort of people with IPF (complete-case analysis)

**Table S3 |** Sensitivity analysis**:** Association between ICS prescribing and pneumonia hospitalisation and all-cause mortality in a cohort of people with IPF (excluding patients who were prescribed ICS only after their IPF diagnosis)

**Table S4a**| Sensitivity analyses: Hazard ratios for the association between ICS prescribing and a) pneumonia hospitalisation and b) all-cause mortality stratified by presence and absence of a comorbid COPD

**Table S4b|** Sensitivity analyses: Hazard ratios for the association between ICS prescribing and a) pneumonia hospitalisation and b) all-cause mortality stratified by presence and absence of a comorbid asthma

**Fig. S1 |** Study design

IPF = Idiopathic pulmonary fibrosis

**Start of study**

01/01/2010

**End of study**

31/12/2019

Index date
(date of IPF diagnosis)

Baseline period (1 year): definition of exposure status

Follow up period for outcomes:
all-cause mortality; hospitalisation for pneumonia

**Fig. S2 |** Flowchart of patients meeting inclusion criteria

CPRD: Clinical Practice Research Datalink; IPF: idiopathic pulmonary fibrosis; UTS: up to standard

| Patients with at least one IPF record in CPRD Aurum and flagged as “regular” and “acceptable”  N = 37,067 | |  |  |
| --- | --- | --- | --- |
|  |  |  | Excluded patients not registered at an  UTS GP practice (n= 362) |
|  |  |  |  |
| IPF patients registered at GP practices which meet  CPRD quality criteria  N = 36,705 | |  |  |
|  |  |  | Excluded patients not registered at a  linked GP practice  (n= 1,556) |
|  |  |  |  |
| IPF patients registered at a linked GP practice  N = 35,149 | |  |  |
|  |  |  | Excluded patients aged under 40 years  at time of diagnosis  (n= 278) |
|  |  |  |  |
| IPF patients aged at least 40 years at the  time of their diagnosis  N = 34,871 | |  |  |
|  |  |  | Excluded patients who received their diagnosis outside the study period (n= 13,323) |
|  |  |  |  |
| Patients diagnosed with IPF between 1/1/2010 and 31/12/2019 (“incident” cases) N = 21,548 | |  |  |
|  |  |  | Excluded patients with less than 12 months  of follow up prior to diagnosis and less than  1 day after diagnosis (n= 4,443) |
|  |  |  |  |
| Total number of patients eligible for inclusion  in study cohort  N = 17,105 | |  |  |

**Table S1** | Patterns of ICS prescribing, pre- and post-IPF diagnosis

| Characteristic | ICS before IPF diagnosis | | | ICS after IPF diagnosis | | | ICS only after IPF diagnosis  (N=1,002) |
| --- | --- | --- | --- | --- | --- | --- | --- |
|  | **Any ICS** (N=4,248) | **Regular**  (N=2,741) | **Irregular**  **(**N**=**1,507**)** | **Any ICS** (N=4,392) | **Regular**  (N=2,961) | **Irregular**  (N=1,431) |  |
| *Age (years)* |  |  |  |  |  |  |  |
| 40–59 | 389 (9.2%) | 241 (8.8%) | 148 (9.8%) | 400 (9.1%) | 270 (9.1%) | 130 (9.1%) | 84 (8.4%) |
| 60–69 | 928 (21.9%) | 570 (20.8%) | 358 (23.8%) | 975 (22.2%) | 654 (22.1%) | 321 (22.4%) | 238 (23.8%) |
| 70–79 | 1,650 (38.8%) | 1,104 (40.3%) | 546 (36.2%) | 1,708 (38.9%) | 1,163 (39.3%) | 545 (38.1%) | 381 (38.0%) |
| 80 and over | 1,281 (30.2%) | 826 (30.1%) | 455 (30.2%) | 1,309 (29.8%) | 874 (29.5%) | 435 (30.4%) | 299 (29.8%) |
| *Gender* |  |  |  |  |  |  |  |
| Male | 2,478 (58.3%) | 170 (61.6%) | 894 (59.3%) | 2,625 (59.8%) | 1,767 (59.7%) | 858 (60.0%) | 659 (65.8%) |
| Female | 1,770 (41.7%) | 106 (38.4%) | 613 (40.7%) | 1,767 (40.2%) | 1,194 (40.3%) | 573 (40.0%) | 343 (34.2%) |
| *Smoking history* | |  |  |  |  |  |  |
| Non-smoker | 391 (9.2%) | 218 (8.0%) | 173 (11.5%) | 389 (8.9%) | 248 (8.4%) | 141 (9.9%) | 101 (10.1%) |
| Ex-smoker | 3,115 (73.3%) | 2,024 (73.8%) | 1,091 (72.4%) | 3,206 (73.0%) | 2,169 (73.3%) | 1,037 (72.5%) | 709 (70.8%) |
| Current smoker | 742 (17.5%) | 499 (18.2%) | 243 (16.1%) | 779 (18.2%) | 544 (18.4%) | 253 (17.7%) | 193 (19.2%) |
| Missing | 0 (0%) | 0 (0%) | 0 (0%) | 0 (0%) | 0 (0%) | 0 (0%) | 0 (0%) |
| *Comorbidities* |  |  |  |  |  |  |  |
| COPD | 1,780 (41.9%) | 1,396 (50.9%) | 384 (25.5%) | 1,750 (39.9%) | 1,306 (44.1%) | 444 (31.0%) | 198 (19.8%) |
| Asthma | 2,143(50.5%) | 1,662(60.6%) | 481 (31.9%) | 1,976(45.0%) | 1,486 (50.2%) | 490 (34.2%) | 116 (11.6%) |

*ICS: inhaled corticosteroid; IPF: idiopathic pulmonary fibrosis*

**Table S2 |** Hazard ratios for the association between PPI/ICS prescribing and pneumonia hospitalisation and all-cause mortality in a cohort of people with IPF (complete-case analysis)

|  |  | **Unadjusted model** | | |  | **Fully-adjusted model^a^** | | |
| --- | --- | --- | --- | --- | --- | --- | --- | --- |
|  |  | **HR** | **95% CI** | **p-value** |  | **HR** | **95% CI** | **p-value** |
| ***Pneumonia hospitalisation*** | | | | | | | | |
| Non-PPI users |  | Reference |  |  |  | Reference |  |  |
| Irregular-PPI users |  | 1.15 | 1.03–1.29 | 0.016 |  | 1.12 | 1.00–1.26 | 0.050 |
| Regular-PPI users |  | 1.19 | 1.10–1.30 | <0.001 |  | 1.14 | 1.04–1.24 | 0.003 |
|  |  |  |  |  |  |  |  |  |
| Non-ICS users |  | 0.00 |  |  |  | 0.00 |  |  |
| Irregular-ICS users |  | 1.15 | 1.03–1.28 | 0.014 |  | 1.19 | 1.06–1.33 | 0.003 |
| Regular-ICS users |  | 1.53 | 1.42–1.66 | <0.001 |  | 1.40 | 1.25–1.55 | <0.001 |
|  | | | | | | | | |
| ***All-cause mortality*** | | | | | | | | |
| Non-PPI users |  | Reference |  |  |  | Reference |  |  |
| Irregular-PPI users |  | 1.05 | 0.86­–1.28 | 0.642 |  | 1.05 | 0.86–1.28 | 0.638 |
| Regular-PPI users |  | 1.06 | 0.91–1.22 | 0.464 |  | 1.04 | 0.90–1.21 | 0.584 |
|  |  |  |  |  |  |  |  |  |
| Non-ICS users |  | 0.00 |  |  |  | 0.00 |  |  |
| Irregular-ICS users |  | 0.89 | 0.83–0.96 | 0.002 |  | 1.02 | 0.95–1.11 | 0.553 |
| Regular-ICS users |  | 1.06 | 1.03–1.15 | 0.002 |  | 1.25 | 1.16–1.34 | <0.001 |

*CI: confidence interval; HR: hazard ratio*

*^a^ Adjusted for age, sex, smoking history, index of multiple deprivation, COPD, asthma, lung cancer, gastro-oesophageal reflux disease, hiatus hernia, ischaemic heart disease, heart failure, pulmonary arterial hypertension and type 2 diabetes. In the case of the PPI analysis, the fully-adjusted model was additionally adjusted for prescription of an oral corticosteroid (at least once in the 12 months prior to IPF diagnosis).*

**Sensitivity analyses**

*BMI*: In light of the high level of missingness in the recording of patients’ BMI (around 30%), our primary analysis did not include BMI as a covariate. Inclusion of BMI in a series of complete–case analyses did not materially alter our effect estimates; we thus concluded that BMI was unlikely to be significant confounder of the association between PPI or ICS prescribing and pneumonia hospitalisation (and all-cause mortality) in this cohort and thus excluded BMI from our subsequent analyses.

*Intention-to-treat/time-varying exposures*: Re-running our Cox models without the 1,002 individuals who were only prescribed ICS after their IPF diagnosis had the effect of increasing our reported hazard ratios, but only by a very small amount. This is to be expected given that inclusion of the patients who initiated ICS after their IPF diagnosis in the control group is likely to bias our estimated HRs towards the null (i.e. dilutes the effect). Excluding these patients from the control group also had a negligible effect on the HRs for all-cause mortality (see **Table S3**). Analysis of ICS prescribing patterns showed that the majority of people who were prescribed ICS prior to IPF diagnosis, continued to do so after. We also explored the people who were hospitalised for pneumonia, the proportion who were still prescribed PPI in the 3 months prior to the hospitalisation. Of the 770 regular PPI users who were hospitalised due to pneumonia, 84.67% were prescribed at least one course of PPI in the three-month period prior to pneumonia hospitalisation.

*Effect modification of selected comorbidities*: We found that neither gastroesophageal reflux disease (p = 0.80) nor hiatus hernia (p = 0.63) were effect modifiers of the association between PPI use and our primary outcome (pneumonia hospitalisation). We found only weak evidence to suggest that concomitant diagnoses of COPD or asthma had a modifying effect on the association between ICS use and pneumonia hospitalisation (or all-cause mortality). In the ICS analyses, the p-values for the interaction terms were all non-significant, save that for the interaction between irregular ICS use and COPD (p=0.049; see **Table S4a**), implying that the risk of pneumonia hospitalisation associated with irregular ICS-use is greater than in those who have COPD than in those who do not have COPD (HR_adj_=1.42 (1.15–1.77) vs 1.10 (0.96–1.26) (relative to non-ICS users).

**Table S3 | Sensitivity analysis:** Association between ICS use and pneumonia hospitalisation and all-cause mortality in a cohort of people with IPF (excluding patients who were prescribed ICS only after their IPF diagnosis)

|  | **Model 1:**  **Unadjusted analysis** | | | **Model 2: Adjusted for demographic and lifestyle factors^a^** | | | **Model 3: Adjusted for demographic  and lifestyle factors^a^ and COPD/asthma** | | | **Model 4:  Fully-adjusted analysis^b^** | | |
| --- | --- | --- | --- | --- | --- | --- | --- | --- | --- | --- | --- | --- |
|  | **HR** | **95% CI** | **P-value** | **HR** | **95% CI** | **P-value** | **HR** | **95% CI** | **p-value** | **HR** | **95% CI** | **P-value** |
| *All-cause mortality* | | | | | | | | | | | | |
| Non-users | Ref. |  |  | Ref. |  |  | Ref. |  |  | Ref. |  |  |
| Irregular users | 0.89 | 0.83–0.96 | 0.003 | 0.99 | 0.92–1.07 | 0.773 | 1.01 | 0.94–1.09 | 0.770 | 1.03 | 0.95–1.12 | 0.438 |
| Regular users | 1.09 | 1.03–1.16 | 0.002 | 1.17 | 1.11–1.24 | <0.001 | 1.23 | 1.14–1.32 | <0.001 | 1.25 | 1.16–1.35 | <0.001 |
| *Hospitalisation for pneumonia* | | | | | | | | | | | | |
| Non-users | Ref. |  |  | Ref. |  |  | Ref. |  |  | Ref. |  |  |
| Irregular users | 1.18 | 1.06–1.32 | 0.003 | 1.29 | 1.15–1.44 | <0.001 | 1.21 | 1.08–1.36 | 0.001 | 1.23 | 1.10–1.39 | <0.001 |
| Regular users | 1.58 | 1.46–1.71 | <0.001 | 1.64 | 1.51–1.78 | <0.001 | 1.43 | 1.28–1.60 | <0.001 | 1.46 | 1.30–1.63 | <0.001 |

*CI: confidence interval; COPD, chronic obstructive pulmonary disease; HR: hazard ratio*

*^a^ Adjusted for age, sex, smoking history and Index of multiple deprivation (IMD)*

*^b^ Adjusted for age, sex, smoking history, index of multiple deprivation, COPD, asthma, lung cancer, gastro-oesophageal reflux disease, hiatus hernia, ischaemic heart disease, heart failure, pulmonary arterial hypertension and type 2 diabetes.*

**Table S4a** **| Sensitivity analysis:**  Hazard ratios for the association between ICS prescribing and a) pneumonia hospitalisation and b) all-cause mortality stratified by presence and absence of comorbid COPD (fully-adjusted model)

|  | 1. **Hospitalisation for pneumonia**   **(weak evidence of an interaction with COPD for irregular users (p= 0.049) but not regular users (p= 0.613)** | | |  | **b) All-cause mortality  (p value for the interaction terms are  not significant)** | | |
| --- | --- | --- | --- | --- | --- | --- | --- |
|  | **HR** | **95% CI** | **P-value** |  | **HR** | **95% CI** | **P-value** |
| *With COPD* | | | | | | | |
| Non-users | 0.00 |  |  |  | 0.00 |  |  |
| Irregular users | 1.42 | 1.15–1.77 | 0.001 |  | 1.14 | 0.98–1.34 | 0.097 |
| Regular users | 1.38 | 1.17–1.63 | *<0.001* |  | 1.33 | 1.19 –1.50 | *<0.001* |
| *Without COPD* | | | | | | | |
| Non-users | 0.00 |  |  |  |  |  |  |
| Irregular users | 1.10 | 0.96–1.26 | 0.164 |  | 0.99 | 0.91–1.09 | 0.902 |
| Regular users | 1.46 | 1.28–1.66 | <0.001 |  | 1.21 | 1.11–1.33 | <0.001 |

*CI: confidence interval; COPD, chronic obstructive pulmonary disease; HR: hazard ratio*

**Table S4b | Sensitivity analysis**: Hazard ratios for the association between ICS prescribing and a) pneumonia hospitalisation and b) all-cause mortality stratified by presence and absence of a comorbid asthma (fully-adjusted model)

|  | 1. **Hospitalisation for pneumonia**   **(p-value for the interaction terms are not significant)** | | |  | **b) All-cause mortality**  **(p-value for the interaction terms are  not significant)** | | | |
| --- | --- | --- | --- | --- | --- | --- | --- | --- |
|  | **HR** | **95% CI** | **P-value** |  | **HR** | **95% CI** | **P-value** | |
| *With asthma* | | | | | | | |  |
| Non-users | 0.00 |  |  |  | 0.00 |  |  |  |
| Irregular users | 1.06 | 0.84–1.35 | 0.608 |  | 1.01 | 0.85–1.19 | 0.912 |  |
| Regular users | 1.26 | 1.05–1.51 | 0.011 |  | 1.20 | 1.06 –1.36 | *0.004* |  |
| *Without asthma* | | | | | | | |  |
| Non-users | 0.00 |  |  |  |  |  |  |  |
| *Irregular users* | 1.21 | 1.06–1.39 | 0.004 |  | 1.02 | 0.93–1.12 | 0.646 |  |
| *Regular users* | 1.45 | 1.28–1.66 | <0.001 |  | 1.27 | 1.17–1.39 | <0.001 |  |

*CI: confidence interval; HR: hazard ratio*
